# Co-occurrence of seagrass vegetation and coral colonies supports unique fish assemblages: a microhabitat-scale perspective

**DOI:** 10.7717/peerj.14466

**Published:** 2022-11-23

**Authors:** Atsushi Nanami

**Affiliations:** Yaeyama Field Station, Coastal and Inland Fisheries Ecosystem Division, Environment and Fisheries Applied Techniques Research Department, Fisheries Technology Institute, Japan Fisheries Research and Education Agency, Ishigaki, Okinawa, Japan

**Keywords:** Fish assemblages, Seagrass bed, Coral colony, Microhabitat, Co-occurrence

## Abstract

Numerous studies have suggested that seagrass beds provide nursery habitats for juvenile fish in both tropical and subtropical regions. Most of these previous studies applied a landscape-scale perspective, in which seagrass beds and coral reefs are treated as independent, homogenous habitats. However, this perspective might overlook the microhabitat-scale perspective within the habitats, for example, the possibility that small-sized hard substrates (*e.g*., coral colonies) within seagrass beds might serve as fish nurseries. The present study aimed to examine the effects of the presence of microhabitats (small-sized coral colonies) within seagrass beds on the structure of fish assemblages. Fieldwork was conducted at Urasoko Bay, Ishigaki Island, Okinawa, Japan. Four habitat zones were selected: (1) seagrass bed with presence of massive coral colonies (SGCO), (2) seagrass bed without coral colonies (SG), (3) sandy bottom (without seagrass vegetation) with massive coral colonies (CO), and (4) sandy bottom without seagrass vegetation or coral colonies (SA). Six 20 m × 2 m line transects were established and monthly underwater observations were conducted between June and October in 2018 and 2019. A cluster analysis, analysis of similarity, and principal component analysis revealed that the fish assemblage at SGCO was significantly different from the other three habitat zones. This was because some fish species (*e.g*., *Ostorhinchusishigakiensis* and *Lutjanus gibbus*) were almost exclusively present at SGCO and rarely seen at CO, SG, and SA. Most individual fish belonging to these species were found on coral colonies at SGCO, suggesting that the co-occurrence of seagrass vegetation and coral colonies is essential for the habitats of these fish species. Although other fish species present at SGCO were also found at SG, three species, *Parupeneus barberinus*, *Stethojulis strigiventer*, and *Lethrinus atkinsoni*, were more abundant at SGCO with some found on coral colonies in this habitat zone. Several fish species that occurred at both SGCO and CO (*e.g*., *Ostorhinchus properuptus*, *Cheilodispterus quinquelineatus*, *Chrysiptera cyanea*, and *Pomacentrus chrysurus*) were more abundant or showed a greater size range at SGCO, suggesting greater survival rates in this habitat zone because of the co-occurrence of seagrass vegetation and coral colonies. This study demonstrated the existence of a unique fish assemblage structure at SGCO. Although the adoption of a landscape-scale perspective (three-dimensional structure of the vegetation) is necessary, a microhabitat-scale perspective that includes the presence of small hard substrates should also be considered to accurately evaluate the nursery function of seagrass beds.

## Introduction

Numerous studies have suggested that seagrass beds serve as nurseries for many fish species in both tropical and subtropical regions (Nagelkerken et al., 2000, 2002; Cocheret de la Morinière et al., 2002; Du et al., 2018). The fish assemblage structure in seagrass beds has also been shown to be different from that in coral reefs (Nakamura & Sano, 2004a; Unsworth et al., 2008; Honda et al., 2013; Du et al., 2020a). Some of these studies consider seagrass beds and coral reefs as distinct habitat zones and suggest that they support different fish assemblages. Many functions of seagrass beds have also been reported, such as providing abundant food supply and refuge space for fish (Heck, Hays & Orth, 2003; Vonk, Christianen & Stapel, 2008; Du et al., 2018) and supporting fishery production at a global scale (Dorenbosch et al., 2006; Unsworth, Nordlund & Cullen-Unsworth, 2019; Carlson et al., 2021).

Most previous studies have applied a landscape-scale perspective treating seagrass beds and coral reefs as separate habitat zones characterized by a homogeneous environment (Du et al., 2020b), although they are ecologically linked to each other (Dorenbosch et al., 2004, 2007; Adams et al., 2006; Gills et al., 2014). Based on this perspective, different habitat zones consist of distinct habitat structures. In particular, seagrass vegetation growing on sandy bottoms forms seagrass beds, and the three-dimensional structure of this vegetation provides both foraging sites and shelter to fish (Hemminga & Duarte, 2000; Heck, Hays & Orth, 2003). Coral colonies with a high structural complexity on sea floors without seagrass vegetation constitute coral reefs and provide habitat, shelter, and foraging sites to fish (Luckhurst & Luckhurst, 1978; Nanami & Nishihira, 2002, 2003; Nanami & Yamada, 2008).

However, the landscape-scale perspective might overlook the microhabitat-scale perspective within a focal habitat zone. For example, small hard substrates (*e.g*., rock, coral colonies, and accumulated dead coral fragments) have often been found within seagrass beds in tropical regions (Nishihira & Suzuki, 2000; Du et al., 2020b). According to Nagelkerken (2009), microhabitats within seagrass beds (*e.g*., small sand patches and coral colonies) might affect the nursery function of seagrass beds. Indeed, Du et al. (2020b) also demonstrated that various microhabitats within seagrass beds (rubble cavities, sand patches, and hard substrates) provide shelter and foraging sites to some fish and invertebrate species, and suggested that the microhabitat-scale perspective should be considered to determine the precise nursery function of seagrass beds.

In the subtropical region of Okinawa, Japan, seagrass beds are often found adjacent to coral-dominated areas (Nakamura & Sano, 2004a; Shibuno et al., 2008). Some seagrass beds have both seagrass vegetation and small-sized hard substrates, such as coral colonies (Nanami & Yamada, 2009a). The co-occurrence of seagrass vegetation and coral colonies might affect the nursery function of the habitat for juvenile fish. Thus, the present study examined the following hypotheses: (1) coral colonies in seagrass beds act as nurseries for juvenile fish, (2) the co-occurrence of seagrass vegetation and coral colonies have synergetic effects on fish assemblage structure, and (3) such synergetic effects support unique fish assemblages. In this study, seagrass beds with small coral colonies were selected as suitable study areas to examine the effects of microhabitats in seagrass beds on fish assemblage structure. Specifically, the fish assemblage structure in seagrass beds with small-sized coral colonies (microhabitat-scale) was compared with the fish assemblage structure in other habitat zones (*i.e*., seagrass bed without coral colonies, sandy bottom with coral colonies, and sandy bottom without seagrass vegetation or coral colonies). This comparison provides new insights into the effects of microhabitats on the fish nursery function of seagrass beds in subtropical regions.

## Materials and Methods

### Study site

**Figure 1:**
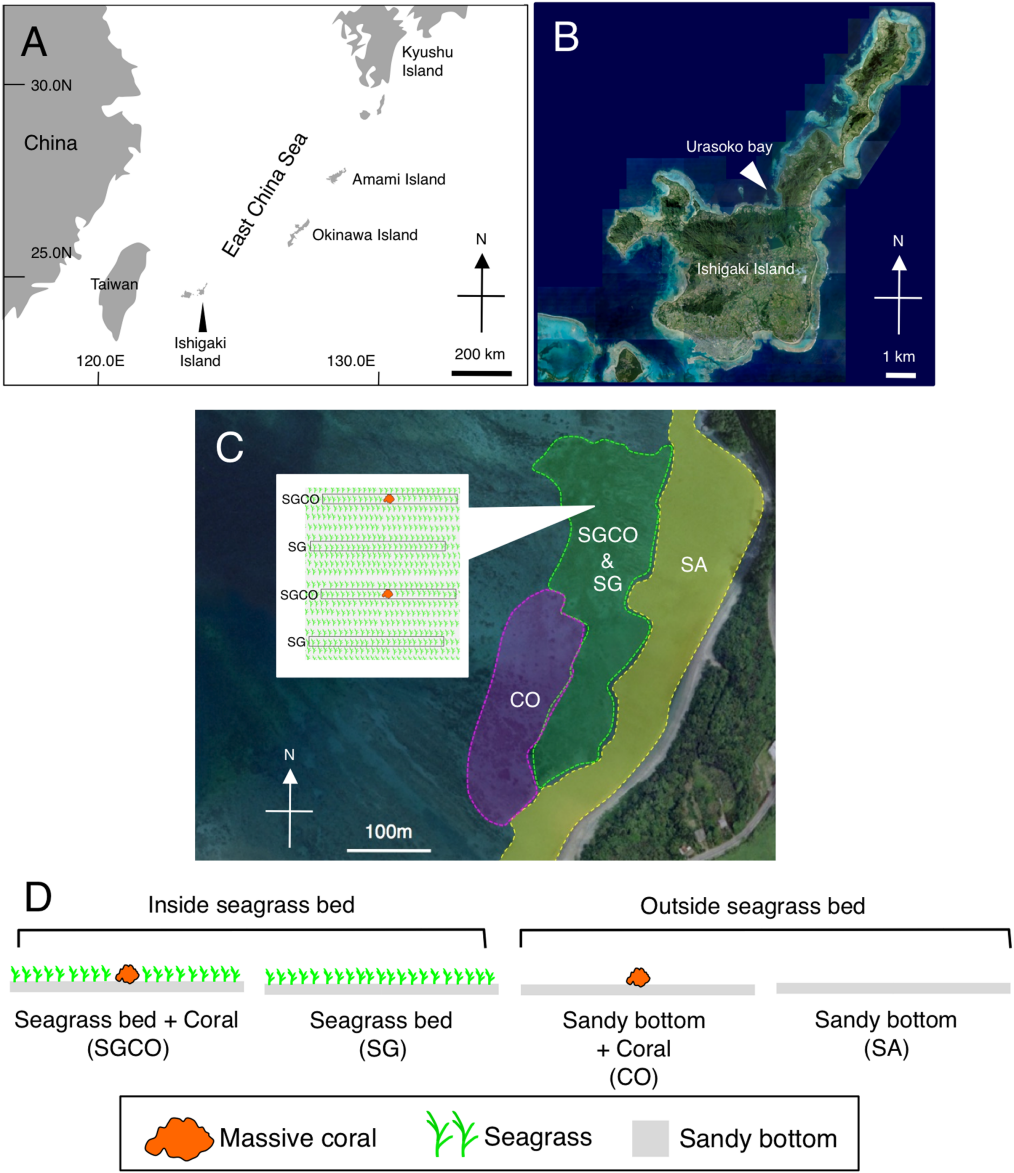
Map of the study site. Showing the location of Ishigaki Island (A) and Urasoko Bay (B). Four habitat zones were selected in this study: (1) seagrass bed with massive coral colonies (SGCO); (2) seagrass bed without coral colonies (SG); (3) sandy bottom (without seagrass vegetation) with massive coral colonies (CO); and (4) sandy bottom without seagrass vegetation or coral colonies (SA). The positions of the four habitat zones are shown in (C). A schematic diagram of SGCO and SG is also shown in (C). A schematic diagram of the four habitat zones is shown in (D). At SGCO and CO, each transect was set so that only one massive coral colony was found at its center. At SG, the positions and directions of transects were set so no coral colonies were included. At SA, the positions and directions of transects were set so no seagrass vegetation or coral colonies were included. The aerial photographs were provided by the International Coral Reef Research and Monitoring Center.

Fieldwork was conducted in Urasoko Bay, Ishigaki Island, Okinawa, Japan (Fig. 1A). An area 450 m × 280 m was selected for the survey (water depth was approximately 2 m at high tide). This area has a sandy bottom, seagrass beds, and coral colonies with a patchy distribution. The seagrass species consisted of *Cymodocea serrulata* and *Thalassia hemprichii*. Most coral species that were found adjacent to seagrass beds were massive corals (*e.g*., *Porites* spp.). Other coral types (*e.g*., branching and tabular corals) are rarely found at shallow depths (Nanami & Yamada, 2009a). Some massive coral colonies of *Porites* were scattered within the seagrass bed.

**Figure 2:**
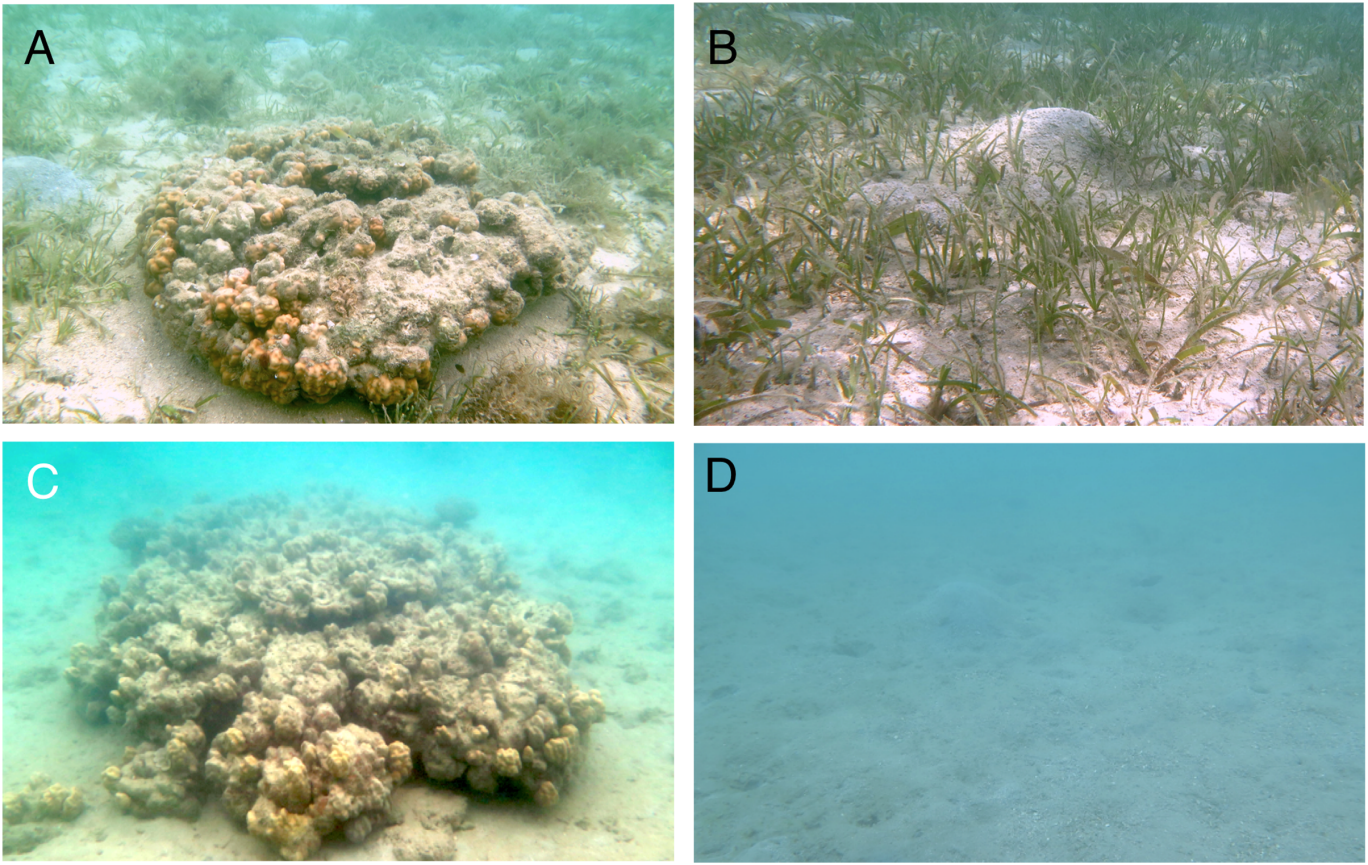
Photographs of the four habitat zones (SGCO, SG, CO and SA). SGCO, seagrass bed with presence of massive coral colony (A); SG, seagrass bed without coral colonies (B); CO, sandy bottom (without seagrass vegetation) with massive coral colony (C); SA, sandy bottom without seagrass vegetation or coral colonies (D).

Four habitat zones were selected (Figs. 1C, 2): (1) homogenous cover of seagrass bed with massive corals (SGCO), (2) homogenous cover of seagrass bed without coral colonies (SG), (3) sandy bottom (without seagrass vegetation) with massive corals (CO), and (4) sandy bottom with no seagrass vegetation or coral colonies (SA).

### Fish survey

Daytime observations (0900–1200) were conducted using six 20 m × 2 m line transects, established in each habitat zone. At SGCO and CO, each transect was set with only one massive coral colony at its center. At SG, each transect was set so as not to include massive coral colonies, and at SA, each transect was set so as not to include seagrass vegetation nor coral colonies. The locations of these transects were fixed throughout the study period, and their start and end points were recorded using a portable GPS receiver to allow repeated observations at the same transects during the study period. At SGCO and SG, the seagrass cover was homogenous inside all transects. Since Nakamura & Sano (2004b) found that the difference in seagrass species did not affect the number of fish species, number of individual fish, or fish species composition, seagrass species composition likely did not affect the fish assemblage structure in the present study. A previous pilot study revealed that juvenile fish settlements occurred predominantly between June and October at the study site (A. Nanami, 2009, pers. comm.). Thus, monthly underwater observations were conducted during these 5 months in 2018 and 2019 for a total of 10 surveys (5 months × 2 years). An observer (A.N.) slowly swam along the transects with great care to avoid scaring the fish. Then, the fish and their estimated total length (TL, 0.5 cm intervals) were recorded. Species identification was performed based on the methods of Masuda & Kobayashi (1994). At SGCO and CO, individual fish found within c.a. 20 cm from the surface of the coral colonies and those further away were recorded separately.

### Coral morphology measurements

Two morphological parameters (diameter and substrate complexity) were measured to verify whether or not the morphological characteristics of massive coral colonies within the SGCO and CO transects were different.

The length of the longest and shortest diameters of each coral colony was measured, and the average values were used for the analysis. Structural complexity was measured as follows: (1) a tape measure was laid to fit the contour of the coral colony surface, and the tape measure length was recorded. In this procedure, the tape measure was laid along the above-mentioned longest and shortest diameters; (2) the tape measure length for the longest and shortest diameters for each colony were averaged; (3): then, substrate complexity was calculated as follows: substrate complexity = average tape measure length/average diameter.

The average diameters of the massive colonies in the transects at SGCO and CO were as follows: SGCO *vs*. CO = 103.1 cm ± 36.8 standard deviation (SD) *vs*. 163.7 cm ± 69.5 SD for average diameter (range: 67.5–157.5 cm *vs*. 67.5–273.0 cm). The average substrate complexities of the massive colonies in the transects at SGCO and CO were as follows: SGCO *vs*. CO =1.51 ± 0.25 SD *vs*. 1.68 ± 0.18 SD for average substrate complexity (range: 1.22–1.87 *vs*. 1.41–1.94). The Mann–Whitney U-test revealed that no significant differences in average diameter and substrate complexity were observed between SGCO and CO, indicating that differences in coral morphology between the two habitat zones were not a cofounding factor on fish assemblage structure in this study.

### Data analysis

This study aimed to examine differences in the number of individual fish and fish species among four habitat zones throughout the study period. Thus, a repeated-measures analysis of variance (RM-ANOVA) was applied. As the assumption of homogeneity of variance was not confirmed, the Games–Howell test was applied for multiple comparisons of the number of individual fish and fish species among the four habitat zones.

A cluster analysis using the group average linkage method with the Bray–Curtis similarity index was performed to determine the spatial and temporal variations in the fish assemblage structures among the four habitat zones. The data obtained from six transects in the same habitat zone during the same month were averaged. A similarity percentage (SIMPER) analysis was also applied to determine the fish species that contributed to the average similarity within a group, which was identified by cluster analysis (Clarke, 1993). In addition, the analysis of similarities (ANOSIM) was applied to evaluate significant differences in fish assemblage structure among the four habitat zones (SGCO, SG, CO, and SA). A principal component analysis (PCA) was also applied to summarize the relationship between the occurrence of each fish species and the four habitat zones.

Although a total of 77 fish species and one genus were found (Table 1), 26 dominant species (each with over 20 individual fish observed over 2 years) were used for the analyses (Table 1). Prior to the analyses, the number of fish was translated to log (x+1). Analyses were performed using the PRIMER (version 6) software package (Clarke & Warwick, 1994).

**Table 1:** List and number of individual fish that were observed at four habitat zones during the present study.

Family	Species	Number of individuals				Total	Analyses	Range of total length (mm)	Growth stage
		SGCO	CO	SG	SA				
Muraenidae	*Echidna nebulosa*	1				1		–*	–*
Holocentridae	*Neoniphon sammara*	3				3		8–14	J
	sp.1	5				5		10	A
Scorpaenidae	*Dendrochirus zebra*		1			1		14	A
	*Pterois volitans*	6	7			13		15–20	J
Epinephelidae	*Epinephelus maculatus*	1				1		7	J
	*Epinephelus merra*		2			2		15	J
	*Epinephelus ongus*	1				1		13	J
Apoginidae	*Cheilodipterus quinquelineatus*	22	74			96	X	2–8	J, A
	*Ostorhinchus ishigakiensis*	1,279		24		1,303	X	2–6	J, A
	*Ostorhinchus properuptus*	1,193	235			1,428	X	2–7	J, A
	*Rhabdamia* sp.1	47				47	X	6–8	J, A
Lutjanidae	*Lutjanus decussatus*		2			2		20	A
	*Lutjanus fulvus*				3	3		3–4	J
	*Lutjanus gibbus*	653			1	654	X	4–12	J
	*Lutjanus kasmira*	29	1			30	X	3.5–12	J
	*Lutjanus quinquelineatus*	10	2		2	14		3–15	J
Caesionidae	*Pterocaesio* sp.1	25	24			49	X	3–6	J
Lethrinidae	*Gnathodentex aureolineatus*	11				11		7–17	J, A
	*Lethrinus atkinsoni*	22		9		31	X	4–10	J
	*Lethrinus harak*	16	12	9	1	38	X	15–30	J, A
	*Lethrinus nebulosus*	11	8	2		21	X	5–20	J
	*Lethrinus obsoletus*	11		11	1	23	X	2–13	J
	*Monotaxis grandoculis*	7	2			9		3.5–5	J
Muliidae	*Mulloidichthys flavolineatus*		166			166	X	12–20	J, A
	*Parupeneus barberinoides*	36		32		68	X	3–10	J
	*Parupeneus barberinus*	65	1	31		97	X	5–20	J, A
	*Parupeneus ciliatus*	13	7			20	X	4–12	J
	*Parupeneus indicus*	3	4			7		5–25	J, A
	*Parupeneus multifasciatus*	49	5	4		58	X	4–15	J, A
	*Parupeneus pleurostigma*	3				3		7	J
	*Upeneus tragula*	1	9			10		12–20	J, A
Chaetodontidae	*Chaetodon auriga*	19	10			29	X	2–20	J, A
	*Chaetodon ephippium*	2				2		3	J
	*Chaetodon vagabundus*	2				2		3	J
	*Heniochus acuminatus*	3				3		3	J
Pomacentridae	*Abudefduf sexfasciatus*	45				45	X	3–5	J, A
	*Abudefduf vaigiensis*	1				1		3	J
	*Chromis viridis*		2			2		1	J
	*Chrysiptera cyanea*	27	20			47	X	2–5	J, A
	*Dascyllus aruanus*	1	5			6		1.5–4	J, A
	*Dascyllus trimaculatus*	10	2			12		2–5	J
	*Dischistodus prosopotaenia*	8	22			30	X	1–20	J, A
	*Neopomacentrus cyanomos*		2			2		2	J
	*Pomacentrus amboinensis*		2			2		2–3	J
	*Pomacentrus chrysurus*	30	12			42	X	1.5–7	J, A
	*Pomacentrus moluccensis*		11			11		1-5	J, A
	*Pomacentrus nagasakiensis*		1			1		4	J
	*Pomacentrus* sp.1	3	130			133	X	1–7	J, A
	*Stegastes* sp.1	4				4		1	J
Polynemidae	sp.1				2	2		20	A
Labridae	*Calotomus spinidens*	1		1		2		10	J
	*Cheilio inermis*	4		3		7		7–11	J
	*Choerodon anchorago*	2	2			4		5–20	J, A
	*Coris batuensis*	2	12			14		1–15	J, A
	*Cymolutes torquatus*	1				1		8	A
	*Halichoeres melanurus*	3	14			17		2–10	J,A
	*Halichoeres trimaculatus*	2				2		10–15	J
	*Iniistius pentadactylus*			1		1		10	A
	*Labroides dimidiatus*		3			3		6–7	J
	*Stethojulis strigiventer*	87		14		101	X	4–10	J, A
	*Scarus ghobban*	1				1		12	J
	*Scarus* spp.	7	1		1	9		3–4	J
Gobiidae	*Amblygobius phalaena*		1		4	5		3–8	J, A
	*Amblyeleotris* sp.1				21	21	X	6–18	J, A
Siganidae	*Siganus fuscescens*	2,417	1	1,842	29	4,289	X	2–7	J
	*Siganus spinus*	3			1	4		4	J
	*Siganus virgatus*	11				11		2–4	J
Acanthuridae	*Acanthurus dussumieri*	1	1			2		8–19	J
	*Acanthurus nigrofuscus*	26	37		1	64	X	2–20	J, A
	*Acanthurus xanthopterus*	16	3			19		2–12	J, A
	*Naso unicornis*	10			2	12		2.5–3.5	J
	sp.1	3				3		4	J
Balistidae	*Balistapus undulatus*		1			1		18	A
	*Balistoides viridescens*		2			2		13–17	J
Ostraciidae	*Ostracion cubicum*		1			1		8	A
Tetraodontidae	*Arothron nigropunctatus*		3			3		15–22	A
Diodontidae	*Chilomycterus reticulatus*				1	1		30	A
Total no. of individuals		6,275	863	1,983	70	9,191			
Total no. of species		57	43	13	13	78			

**Notes:**

* Total length could not be estimated since whole body of the individual could not be observed.

SGCO, seagrass bed with coral colonies; CO, sandy bottom with coral colonies; SG, seagrass bed without coral colonies; SA, sandy bottom without seagrass vegetation or coral colonies. For growth stage, J, juveniles; A, adults.

### Size frequency

The habitat zone-specific size frequency was plotted to examine differences in size frequency among habitat zones for fish species detected at SGCO (fish species occurring at SGCO only: at SGCO and SG; at SGCO and CO; and at SGCO, SG, and CO: see Results). Individual fish found on coral colonies, in the seagrass vegetation, and on the sandy bottom at SGCO and CO were all plotted separately.

## Results

### Fish fauna

A total of 9,191 individual fish, representing 77 fish species and one genus (*Scarus*), were observed during the study period (Table 1). A total of 6,275 individual fish (57 species and one genus, *Scarus*) were observed at SGCO, 863 individual fish were observed at CO (43 species and one genus, *Scarus*), 1,983 individual fish were observed at SG (13 species), and 70 individual fish were observed at SA (13 species and one genus, *Scarus*). The most dominant species were *Siganus fuscescens*, *Ostorhinchus properuptus, O. ishigakiensis*, and *Lutjanus gibbus* (Fig. S1), which combined accounted for 83.5% of the total individual fish observed (7,674 individual fish in total for the four species).

### Number of individual fish and fish species

**Figure 3:**
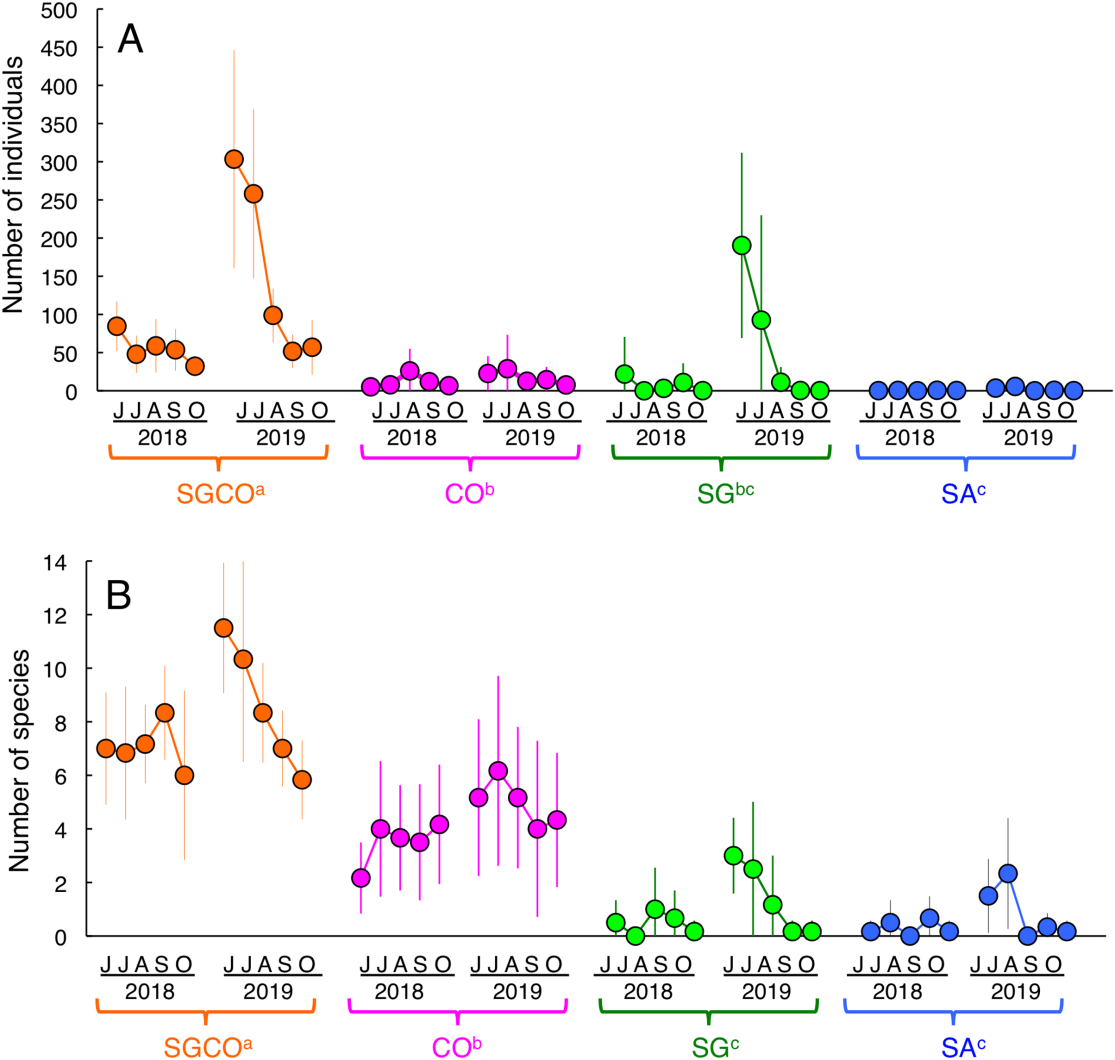
Seasonal and annual variations in the average number of individual fish and fish species during the study period. Four habitat zones are shown as abbreviations: SGCO, seagrass bed with massive coral colonies; SG, seagrass bed without coral colonies; CO, sandy bottom (without seagrass vegetation) with massive coral colonies; and SA, sandy bottom without seagrass vegetation or coral colonies. Different colors represent different habitat zones (orange, SGCO; magenta, CO; green, SG; blue, SA). Bars represent standard deviations. Different letters next to habitat zones (SGCO, CO, SG, and SA) represent significant differences in the number of individual fish or fish species obtained from the Games–Howell multiple comparison test (*p* < 0.05).

An RM-ANOVA revealed that the number of individual fish at SGCO was significantly greater than in the other three habitat zones (Fig. 3A). The number of individual fish at CO was also significantly greater than that at SA, but not significantly different from the number of fish at SG. No significant differences were found between SG and SA.

The number of species at SGCO was also significantly greater than in the other three habitat zones (Fig. 3B). The number of species at CO was also significantly greater than at SG and SA, but no significant differences were found between SG and SA.

### Spatial and monthly variations in fish assemblage structure

**Figure 4:**
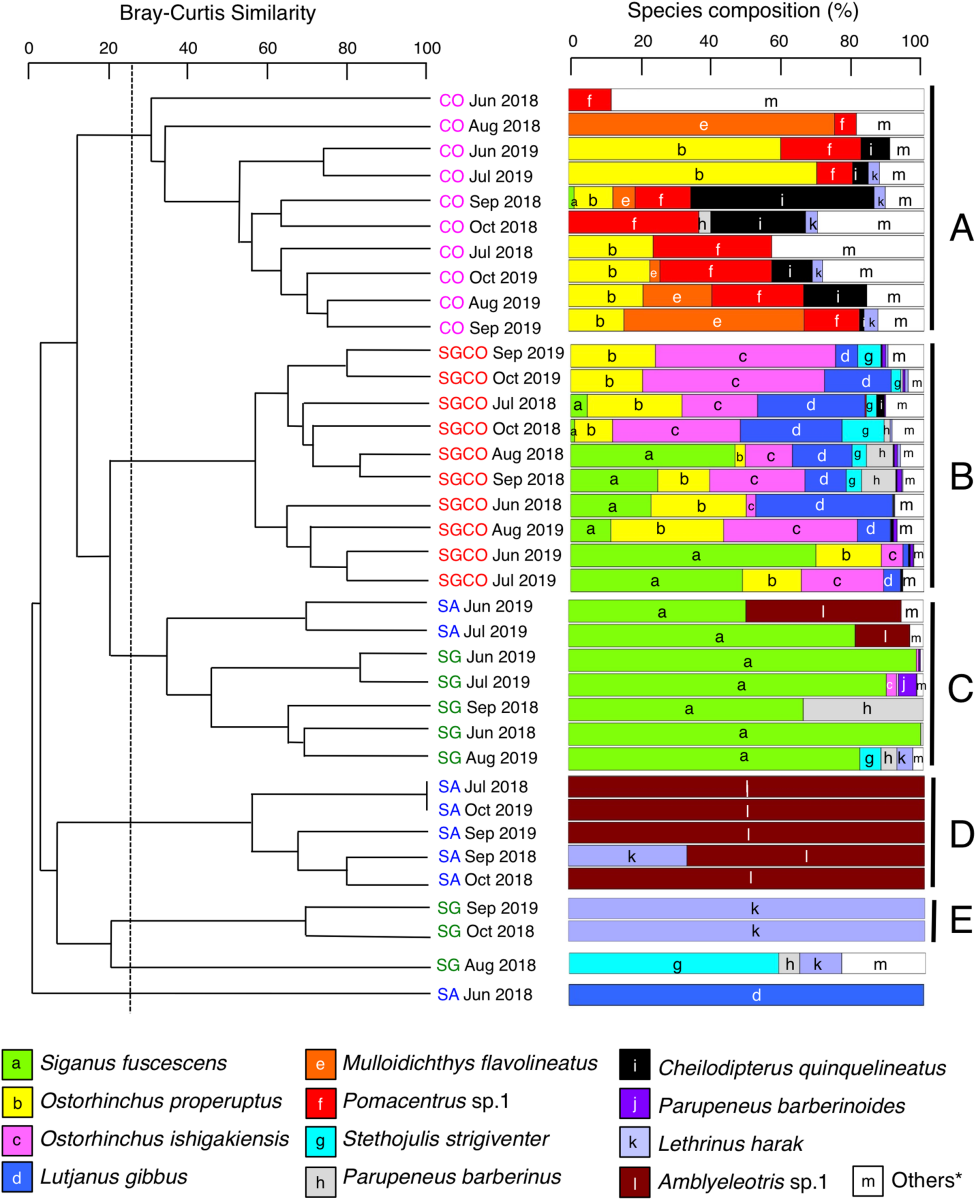
Dendrogram of hierarchical clusters. Representing the fish assemblage structure obtained *via* the group-average-linkage method using the Bray–Curtis similarity index (A), and actual species composition of the fish assemblages (B). The fish data obtained from six transects within the same habitat zone were averaged for each month. Log (x+1)-transformed fish abundance data was used in the analysis. The fish assemblage structure was divided into five groups (A–E) at a 25% similarity level. Four habitat zones are shown as abbreviations: SGCO, seagrass bed with massive coral colonies; SG, seagrass bed without coral colonies; CO, sandy bottom (without seagrass vegetation) with massive coral colonies; and SA, sandy bottom without seagrass vegetation or coral colonies; and *, “Others” includes 14 species (*Abudefduf sexfasciatus*, *Acanthurus nigrofuscus*, *Chaetodon auriga*, *Chrysiptera cyanea*, *Dischistodus prosopotaenia*, *Lethrinus atkinsoni*, *Lethrinus nebulosus*, *Lethrinus obsoletus*, *Lutjanus kasmira*, *Parupeneus ciliatus*, *Parupeneus multifasciatus*, *Pomacentrus chrysurus*, *Pterocaesio* sp.1, and *Rhabdamia* sp.1). Since no fish were observed during two observations at SG (July 2018 and October 2019) and SA (August 2018 and August 2019), these observations were excluded from the analyses.

A cluster analysis revealed that fish assemblages could be divided into five groups: Groups A and B consisted of all 10 months at CO and SGCO, respectively (Fig. 4); Group C consisted of 5 months at SG and 2 months at SA; Group D consisted of 5 months at SA; and group E consisted of 2 months at SG. Overall, the ANOSIM revealed significant differences in fish assemblage structures among the four habitat zones (*p* < 0.001 for all pairs among four habitat zones).

A SIMPER analysis revealed the dominant species in each group (Table 2). Eight species were categorized as dominant in group A, three of which (*Pomacentrus* sp.1, *Ostorhinchus properuptus* and *Acanthurus nigrofuscus*) accounted for over 10% of the similarity of the group. Eleven species were dominant in group B, with four species accounting for over 10% of group similarity (*Ostorhinchus ishigakiensis, O. properuptus, Lutjanus gibbus* and *Siganus fuscescens*). In group C, *Siganus fuscescens* was the dominant species (accounting for 88.96% of the group similarity), followed by *Parupeneus barberinus* (4.55%). In contrast, *Amblyeleotris* sp.1 was the only species identified in group D and *Lethrinus harak* was the only species identified in group E.

**Table 2:** Results of the similarity percentage analysis (SIMPER analysis) for 26 dominant species.

Species	Average total number at each month	Average similarity	Contribution to average similarity (%)
Group A (average similarity = 48.18)			
*Pomacentrus* sp.1	1.10	16.92	35.12
*Ostorhinchus properuptus*	1.04	7.85	16.29
*Acanthurus nigrofuscus*	0.44	5.08	10.54
*Cheilodipterus quinquelineatus*	0.62	4.79	9.95
*Dischistodus prosopotaenia*	0.30	3.90	8.10
*Chrysiptera cyanea*	0.26	2.43	5.05
*Mulloidichthys flavolineatus*	0.67	2.37	4.92
*Pomacentrus chrysurus*	0.17	1.53	3.19
			
Group B (average similarity = 62.79)			
*Ostorhinchus ishigakiensis*	2.82	14.71	23.43
*Ostorhinchus properuptus*	2.66	12.88	20.51
*Lutjanus gibbus*	2.32	12.75	20.30
*Siganus fuscescens*	2.30	6.47	10.30
*Stethojulis strigiventer*	0.74	2.75	4.38
*Pomacentrus chrysurus*	0.40	2.16	3.45
*Parupeneus multifasciatus*	0.50	1.43	2.28
*Parupeneus barberinoides*	0.40	1.17	1.86
*Chaetodon auriga*	0.27	1.14	1.81
*Parupeneus barberinus*	0.49	1.03	1.64
*Lethrinus atokinsoni*	0.29	0.95	1.51
			
Group C (average similarity = 46.23)			
*Siganus fuscescens*	2.79	41.13	88.96
*Parupeneus barberinus*	0.38	2.10	4.55
			
Group D (average similarity = 64.71)			
*Amblyeostris* sp.1	0.28	64.71	100
			
Group E (average similarity = 69.78)			
*Lethrinus harak*	0.22	69.78	100

### Relationship between the occurrence of each fish species and the four habitat zones

**Figure 5:**
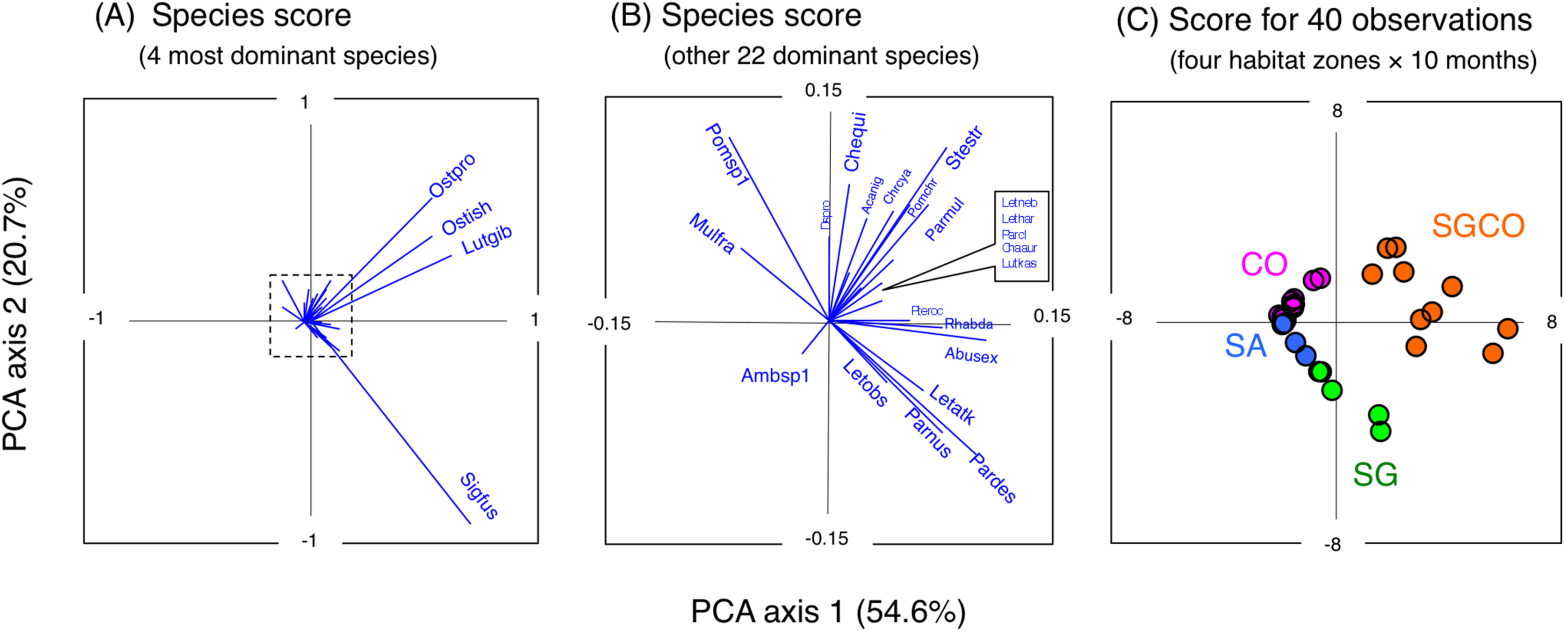
Results of principal component analysis (PCA). Indicating the relationship between the occurrence of 26 fish species (A, B) and 40 observations ((C) (four habitat zones × 10 months)). In (A) and (B), the occurrence of each fish species is shown by abbreviations as follows. Abusex, *Abudefduf sexfasciatus*; Acanig, *Acanthurus nigrofuscus*; Ambsp1, *Amblyeleotris* sp.1.; Chaaur, *Chaetodon auriga*; Chequi, *Cheilodipterus quinquelineatus*; Chrcya, *Chrysiptera cyanea*; Dispro, *Dischistodus prosopotaenia*; Letatk, *Lethrinus atkinsoni*; Lethar, *Lethrinus harak*; Letneb, *Lethrinus nebulosus*; Letobs, *Lethrinus obsoletus*; Lutgib, *Lutjanus gibbus*; Lutkas, *Lutjanus kasmira*; Mulfla, *Mulloidichthys flavolineatus*; Ostpro, *Ostorhinchus properuptus*; Ostish, *Ostorhinchus ishigakiensis*; Parcil, *Parupeneus ciliatus*; Pardes, *Parupeneus barberinoides*; Parmul, *Parupeneus multifasciatus*; Parnus, *Parupeneus barberinus*; Pomchr, *Pomacentrus chrysurus*; Pomsp1, *Pomacentrus* sp.1; Pteroc, *Pterocaesio* sp.1; Rhabda, *Rhabdamia* sp.1; Sigfus, *Siganus fuscescens*; Stestr, *Stethojulis strigiventer*.

**Figure 6:**
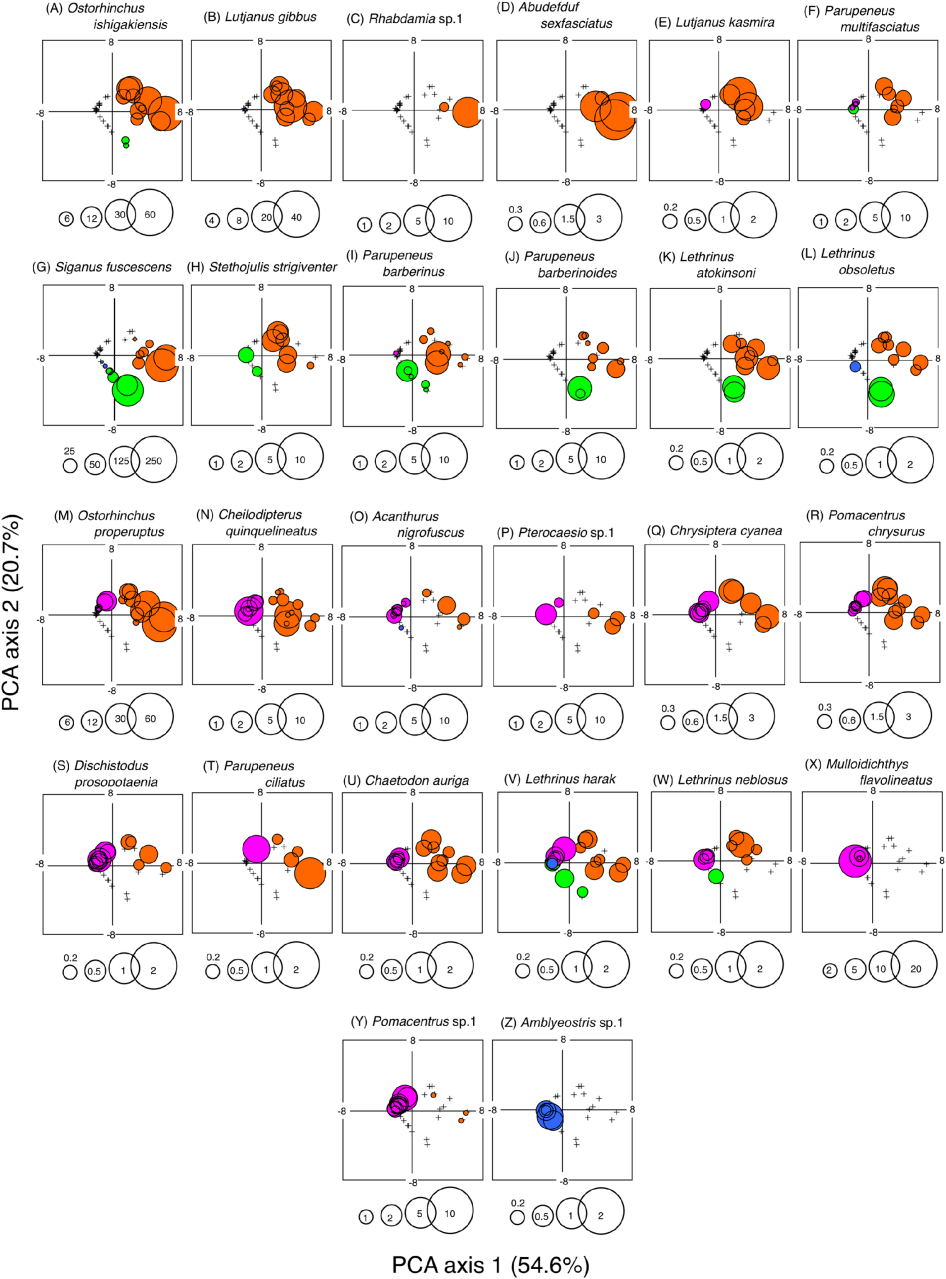
Bubble plot of the PCA results. Indicating the relationship between the abundance of 26 dominant fish species and 40 observations (four habitat zones × 10 months). The bubble plots for each species are shown as overlays on the PCA scores for 40 observations (see also Fig. 5C). The bubble sizes are proportionate to fish abundance. Cross marks represent the absence of individual fish. Four habitat zones are shown as abbreviations: SGCO, seagrass bed with massive coral colonies; SG, seagrass bed without coral colonies; CO, sandy bottom (without seagrass vegetation) with massive coral colonies; and SA, sandy bottom without seagrass vegetation or coral colonies. Different colors represent different habitat zones (orange, SGCO; green, SG; magenta, CO; blue, SA).

A PCA revealed the relationship between the four habitat zones and the species-specific occurrence of the 26 dominant fish species (Figs. 5 and 6). Six types of relationships were found: (1) six species were almost exclusively found at SGCO (*Ostorhinchus ishigakiensis*, *Lutjanus gibbus*, *Rhabdamia* sp.1, *Abudefduf sexfasciatus*, *Lutjanus kasmira*, and *Parupeneus multifasciatus*; Figs. 6A–6F); (2) six species were observed primarily at SGCO and SG (*Siganus fuscescens*, *Stethojulis strigiventer*, *Parupeneus barberinus*, *Parupeneus barberinoides*, *Lethrinus atkinsoni*, and *L. obsoletus*; Figs. 6G–6L); (3) nine species were found primarily at SGCO and CO (*Ostorhinchus properuptus*, *Cheilodipterus quinquelineatus*, *Acanthurus nigrofuscus*, *Pterocaesio* sp., *Parupeneus ciliatus, Chrysiptera cyanea*, *Pomacentrus chrysurus*, *Dischistodus prosopotaenia*, and *Chaetodon auriga*; Figs. 6M–6U), (4) two species were observed primarily at SGCO, SG, and CO (*Lethrinus harak* and *L. nebulosus*; Figs. 6V, 6W); (5) two species were almost exclusively found at CO (*Mulloidichthys flavolineatus* and *Pomacentrus* sp.1; Figs. 6X, 6Y); and (6) one species was only observed at SA (*Amblyeleostris* sp.1; Fig. 6Z).

### The size frequency of fish species observed almost exclusively at SGCO

**Figure 7:**
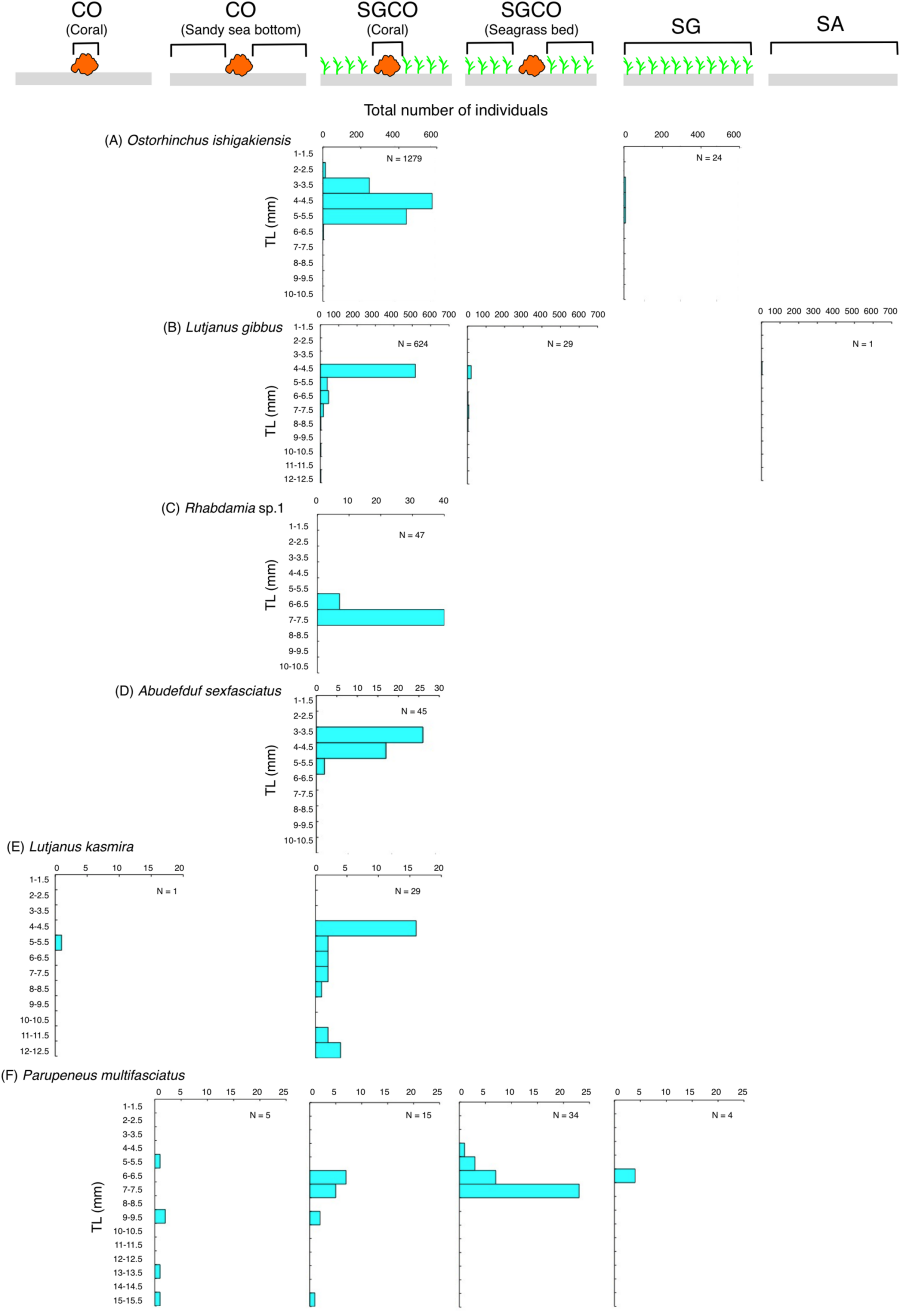
Size frequency of the six fish species that were observed almost exclusively at the seagrass bed with massive coral colonies (SGCO). Size frequencies of individual fish at SGCO on coral colonies are shown separately from individual fish at SGCO in the seagrass vegetation.

Five of the six species (*i.e*., *Ostorhinchus ishigakiensis*, *Lutjanus gibbus*, *Rhabdamia* sp.1, *Abudefduf sexfasciatus*, and *Lutjanus kasmira*) observed mainly at SGCO were almost exclusively found on coral colonies (Figs. 7A–7E). Most individual fish observed of these five species were under 8 cm in length.

In contrast, most of the individual *Parupeneus multifasciatus* fish were found at SGCO, but a greater number of these fish were observed in the seagrass vegetation than on coral colonies. Individual *P. multifasciatus* fish 4–7 cm in length were found in the seagrass vegetation at SGCO, whereas those 5–15 cm in length were found on coral colonies at SGCO and on the sandy bottom at CO (Fig. 7F).

### Size frequency of fish species at SGCO and SG

**Figure 8:**
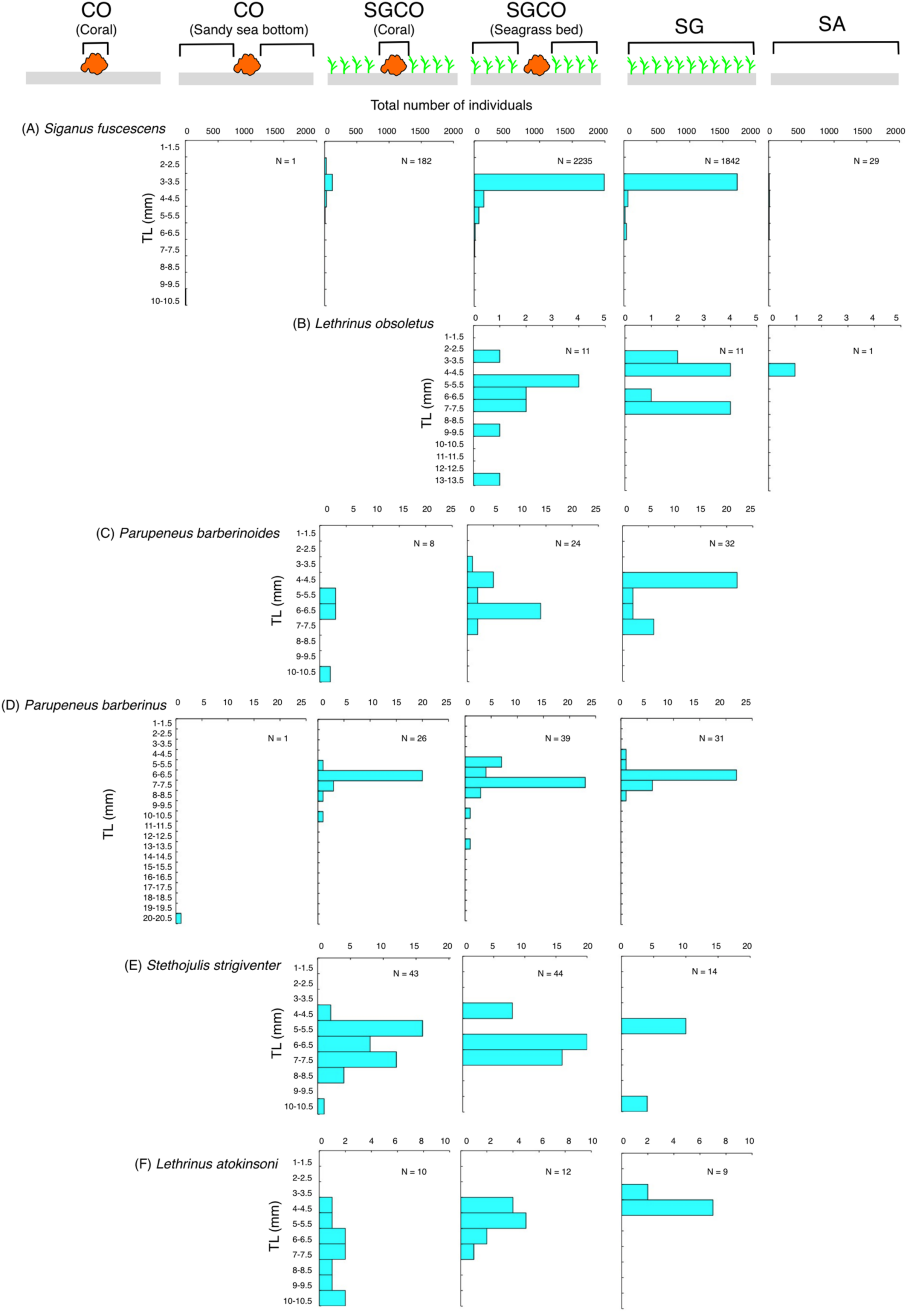
(A–F) Size frequency of the six fish species observed at both the seagrass bed with massive coral colonies (SGCO) and the seagrass bed without coral colonies (SG). Size frequencies of individual fish at SGCO and CO observed on coral colonies, in the seagrass vegetation, and on the sandy bottom are all shown separately.

Among the six species detected at SGCO and SG, most individual *Siganus fuscescens, Lethrinus obsoletus*, and *Parupeneus barberinoides* fish were found in the seagrass vegetation at both SGCO and SG (Figs. 8A–8C). No clear size range differences were observed in these three species between SGCO and SG.

The number of individual *Parupeneus barberinus* and *Stethojulis strigiventer* fish at SGCO was greater than at CO. At SGCO, individual fish were found on both coral colonies and in seagrass vegetation (Figs. 8D, 8E). No clear size range differences were found between SGCO and SG.

More individual *Lethrinus atkinsoni* fish were also observed at SGCO (Fig. 8F). For this species, differences in size range were observed between individual fish at SG (3–4 cm), in the seagrass vegetation at SGCO (4–7 cm), and on coral colonies at SGCO (4–10 cm).

### Size frequency of fish species at SGCO and CO

**Figure 9:**
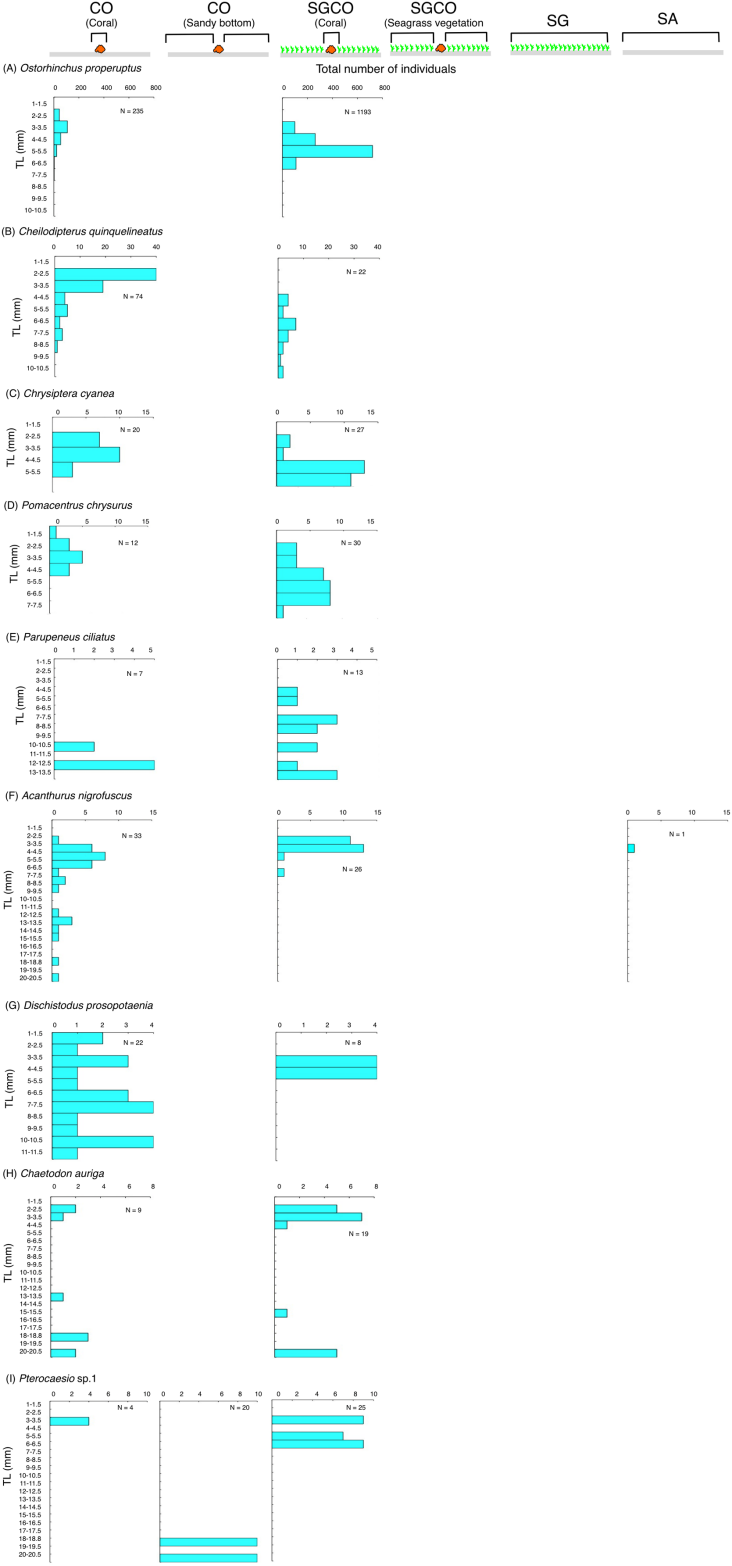
(A–I) Size frequency of the nine fish species observed at both the seagrass bed with massive coral colonies (SGCO) and the sandy bottom (without seagrass vegetation) with massive coral colonies (CO). Size frequencies of individual fish at SGCO and CO on coral colonies, in the seagrass vegetation, and on the sandy bottom are all shown separately.

Of the nine fish species observed at both SGCO and CO, more individual *Ostorhinchus properuptus* fish were found at SGCO, though individual *Ostorhinchus properuptus* fish were found on coral colonies at both SGCO and CO. No clear size range differences were found between the two habitat zones (Fig. 9A).

Three species (*Cheilodipterus quinquelineatus*, *Chrysiptera cyanea*, and *Pomacentrus chrysurus*) that were found on coral colonies at both SGCO and CO had a slightly larger size range at SGCO than at CO (Figs. 9B–9D).

Although individual *Parupeneus ciliatus* fish were found on coral colonies at SGCO and CO, individual fish 4–10 cm in length were only found at SGCO (Fig. 9E).

Most individual *Acanthurus nigrofuscus* and *Dischistodus prosopotaenia* fish were found on coral colonies at both SGCO and CO, but individual fish 12–20 cm in length were predominantly found at CO (Figs. 9F, 9G).

There was no clear difference in the size range of *Chaetodon auriga* fish between SGCO and CO (Fig. 9H).

Smaller-sized individual *Pterocaesio* sp.1 fish (3–6 cm) were found on coral colonies at both SGCO and CO. In contrast, larger-sized individual *Pterocaesio* sp.1 fish (18, 20 cm) were only found on the sandy bottom at CO (Fig. 9I).

### Size frequency of fish species at SGCO, CO, and SG

**Figure 10:**
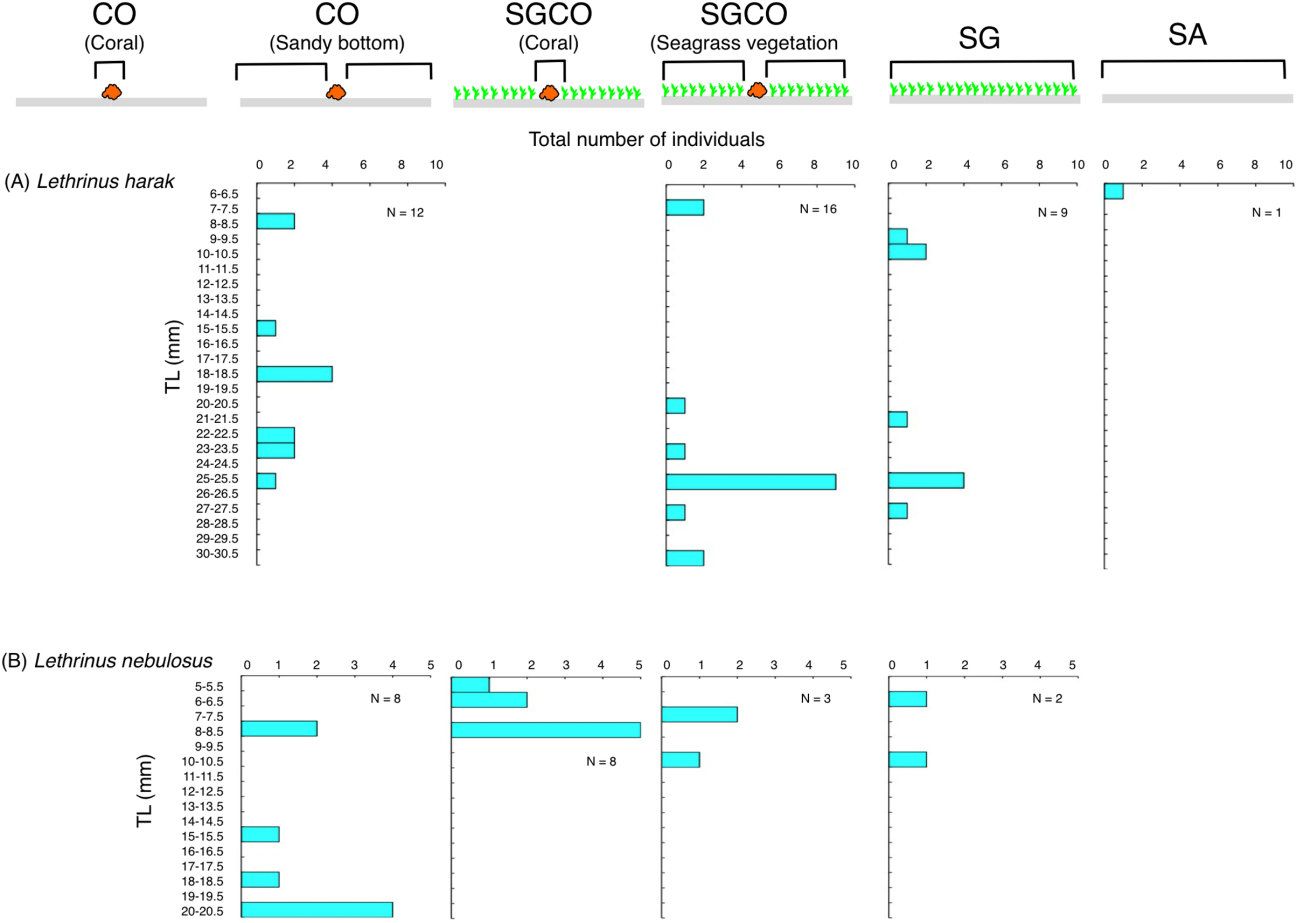
(A–B) Size frequency of the two fish species observed at the seagrass bed with massive coral colonies (SGCO), the seagrass bed without coral colonies (SG), and the sandy bottom (without seagrass vegetation) with massive coral colonies (CO). Size frequencies of individual fish at SGCO and CO on coral colonies, in the seagrass vegetation, and on the sandy bottom are all shown separately.

Individual *Lethrinus harak* fish were predominantly found in seagrass vegetation and on the sandy bottom at SGCO, CO, and SG, and there were no clear size range differences between the three habitat zones (Fig. 10A). Smaller-sized *Lethrinus nebulosus* fish were found at SGCO (6–11 cm) and SG (7–11 cm), whereas larger-sized individual *Lethrinus nebulosus* fish were observed at CO (8–20 cm; Fig. 10B).

## Discussion

### Unique fish assemblage at SGCO

This study aimed to examine the effects of microhabitats within seagrass beds on fish assemblage structure. The results showed that the fish assemblage structure in seagrass beds differed depending on the presence or absence of massive coral colonies. One reason for this is that two fish species (*Ostorhinchus ishigakiensis* and *Lutjanus gibbus*) were almost exclusively observed at SGCO. A SIMPER analysis revealed that these two fish species accounted for 43.73% of the fish assemblage structure at SGCO (23.43% and 20.30% for *O. ishigakiensis* and *L. gibbus*, respectively). Considering these two species were rarely observed at SG and CO and that most individual fish of these species were found on coral colonies, the co-occurrence of seagrass vegetation and coral colonies is likely essential for their habitat.

The size range of *O. ishigakiensis* at SGCO was 2–6 cm. Since 6 cm is the maximum size of this species (Froese & Pauly, 2022), it is likely that the *O. ishigakiensis* fish observed at SGCO included both juveniles and adults. Conversely, the observed size range of *L. gibbus* at SGCO was 4–8 cm. The maximum total length of this species is over 30 cm (Nanami et al., 2010; Taylor et al., 2018), so the individuals found at SGCO were likely newly-settled juveniles. Since harpacticoid copepods and small benthic animals are the main food sources consumed by *O. ishigakiensis* and newly-settled *L. gibbus* juveniles (Nakamura et al., 2003, 2006; A. Nanami, 2008, pers. comm.), and these prey items are abundant in seagrass beds (Nakamura & Sano, 2005), it is hypothesized that these two fish species at SGCO utilized coral colonies as a refuge space or habitat, and seagrass vegetation as their foraging sites. At SGCO, newly-settled *L. gibbus* juveniles settled directly on the massive corals within the seagrass bed (Nanami & Yamada, 2009a). Thus, the synergetic effect between seagrass vegetation and coral colonies at SGCO might support the presence of these two fish species. In addition, as *L. gibbus* is a main fishery target in coral reefs in worldwide (Akita et al., 2016; Nanami et al., 2010; Taylor et al., 2018), seagrass beds that include coral colonies might be important nurseries for fishery production.

A similar trend was also observed for other three fish species (*Rhabdamia* sp.1, *Abudefduf sexfasciatus* and *Lutjanus kasmira*), suggesting that the co-occurrence of seagrass vegetation and massive coral colonies might also be a suitable environment for these fish species.

Individual *Parupeneus multifasciatus* fish were found in both seagrass vegetation and on coral colonies. As this species is diurnal, mobile, and benthic animal feeders, it is suggested that this fish species utilizes seagrass vegetation as foraging sites and coral colonies as a refuge site, so the co-occurrence of seagrass vegetation and coral colonies might also represent a suitable environment for this species.

### Effects of coral colonies within the seagrass bed on fish species at SGCO and SG

Some fish species found at both SGCO and SG were more abundant at SGCO than at SG (*e.g*., *Parupeneus barberinus*, *Stethojulis strigiventer* and *Lethrinus atkinsoni*), with individual fish found on coral colonies and in seagrass vegetation at SGCO. These species might use coral colonies as a refuge space and seagrass vegetation as a feeding site, since their prey are benthic invertebrates (Nakamura et al., 2003). Smaller-sized individual *L. atkinsoni* fish were found at SG, whereas larger-sized individuals were observed on coral colonies at SGCO. This finding might be due to the direct settlement of pelagic larvae on the seagrass vegetation, which became juveniles (size range = 3–4 cm). As they grow, they might use coral colonies as refuge, as was observed in the larger-sized juveniles (8–10 cm).

Conversely, most *Siganus fuscescens*, *Lethrinus obsoletus*, and *Parupeneus barberinoides* individuals were found in the seagrass vegetation. Some previous studies have reported similar trends (Kanashiro, Motonaga & Kimura, 1999; Shibuno et al., 2008). Therefore, the presence of coral colonies at SGCO did not greatly contribute to the occurrence of these species, suggesting that not all fish species in seagrass beds benefit from coral colonies within those seagrass beds.

### Effects of coral colonies within seagrass beds on fish species at SGCO and CO

Most of the individual fish of the species observed at both SGCO and CO were found on coral colonies. Among them, apogonids and pomacentrids were only found on coral colonies, which is consistent with a previous study (Nanami & Nishihira, 2003). However, individual *Ostorhinchus properuptus* fish were more abundant at SGCO than at CO. In addition, the maximum fish size observed for three species (*Cheilodipterus quinquelineatus*, *Chrysiptera cyanea* and *Pomacentrus chrysurus*) was slightly greater at SGCO than at CO. This might be because of greater survival rates on coral colonies at SGCO than at CO. The co-occurrence of seagrass vegetation and coral colonies might increase the survival rate of fish, since seagrass vegetation and coral colonies provide a three-dimensional structure (Hemminga & Duarte, 2000; Heck, Hays & Orth, 2003) and complex physical structure (Luckhurst & Luckhurst, 1978; Nanami & Nishihira, 2002), respectively. Moreover, smaller-sized individual *Parupeneus ciliatus* fish were found on coral colonies at SGCO whereas larger-sized individuals were found at CO, indicating that this species might also utilize coral colonies within seagrass beds as their habitat.

However, other fish species (*Acanthurus nigrofuscus*, *Dischistodus prosopotaenia*, *Chaetodon auriga* and *Precaesio* sp.1) did not show the same trend, suggesting that not all fish species inhabiting coral colonies benefit from the co-occurrence of seagrass vegetation and coral colonies.

### Effects of coral colonies within seagrass beds on fish species at SGCO, SG, and CO

Almost all individual *Lethrinus harak* fish were found in seagrass vegetation and on the sandy bottom, not on coral colonies. Since this species is diurnal, mobile, and benthic animal feeders (Nanami & Yamada, 2009b), *L. harak* fish swim along the sea floor to search for prey that inhabit the seagrass vegetation and sandy bottom. *Lethrinus nebulosus* showed a similar trend, although some individual fish were found on coral colonies at SGCO. However, as other individual fish were observed in the seagrass vegetation and on the sandy bottom, there is no direct evidence that juveniles preferentially utilize coral colonies within seagrass beds as their habitat. Therefore, these two species do not necessarily benefit from coral colonies within seagrass beds.

### Potential mechanisms of fish settlement on coral colonies within seagrass beds

As previously discussed, some fish species were observed almost exclusively at SGCO (*e.g*., *Ostorhinchus ishigakiensis* and *Lutjanus gibbus*). This might be because the combination of seagrass vegetation and corals provides the most suitable environment for these species. Pelagic larvae of *L. gibbus* use chemical cues originating from seagrass beds to select the location in which to settle (Lecchini & Nakamura, 2013), directly settling on massive corals within those seagrass beds (Nanami & Yamada, 2009a). Since seagrass beds support abundant prey for juveniles and coral colonies provide a complex physical structure, this settlement behavior might be explained by the need for sufficient prey and a suitable refuge space. Whether a similar behavior occurs in other fish species observed at SGCO remains unclear, but the habitat structure of both seagrass vegetation and coral colonies, and chemical cues originating from them might be the essential factors in the selection of SGCO as a suitable environment by these fish species.

## Conclusions

The results of this study suggest that seagrass beds that include microhabitats (*i.e*., coral colonies) support a unique fish assemblage structure compared with seagrass beds that do not. Microhabitats within seagrass beds provide a unique combination of habitat, refuge space, and foraging ground. This result is supported by the observed occurrence of unique fish species in the seagrass bed with coral colonies. Although several other fish species were found at SGCO, SG, and CO, some fish species might specifically benefit from the presence of microhabitats within seagrass beds. As seagrass beds in other regions likely consist of both seagrass vegetation and various types of microhabitats (*e.g*., smaller-sized coral colonies, accumulation of coral fragments and rocks), the importance of these microhabitats, which serve as nurseries within seagrass beds, should be examined in more detail. According to Nagelkerken (2009) and Du et al. (2020b), such examination would clarify the importance of the presence of microhabitats as fish nurseries in seagrass beds in both tropical and subtropical regions.

## Supplemental Information

10.7717/peerj.14466/supp-1 Supplemental Information 1 Total number of individuals for each species detected during the study period. Red lines represent thresholds between dominant (over 20 individuals) and non-dominant species (less 19 individuals).

10.7717/peerj.14466/supp-2 Supplemental Information 2 Figure 3 Raw data.

10.7717/peerj.14466/supp-3 Supplemental Information 3 Figure 4 Raw data.

10.7717/peerj.14466/supp-4 Supplemental Information 4 Figure 5 Raw data.

10.7717/peerj.14466/supp-5 Supplemental Information 5 Figure 6 Raw data.

10.7717/peerj.14466/supp-6 Supplemental Information 6 Figure 7 Raw data.

10.7717/peerj.14466/supp-7 Supplemental Information 7 Figure 8 Raw data.

10.7717/peerj.14466/supp-8 Supplemental Information 8 Figure 9 raw data.

10.7717/peerj.14466/supp-9 Supplemental Information 9 Figure 10 Raw data.

## Additional Information and Declarations

### Data Availability

The following information was supplied regarding data availability: The raw data is available in the Supplemental Files.
