# Supplementary figures and images for: Co-occurrence of seagrass vegetation and coral colonies supports unique fish assemblages: a microhabitat-scale perspective
Source: PeerJ. 2022 Nov 23;10:e14466. doi: 10.7717/peerj.14466 (PMC9700455; doi:10.7717/peerj.14466)

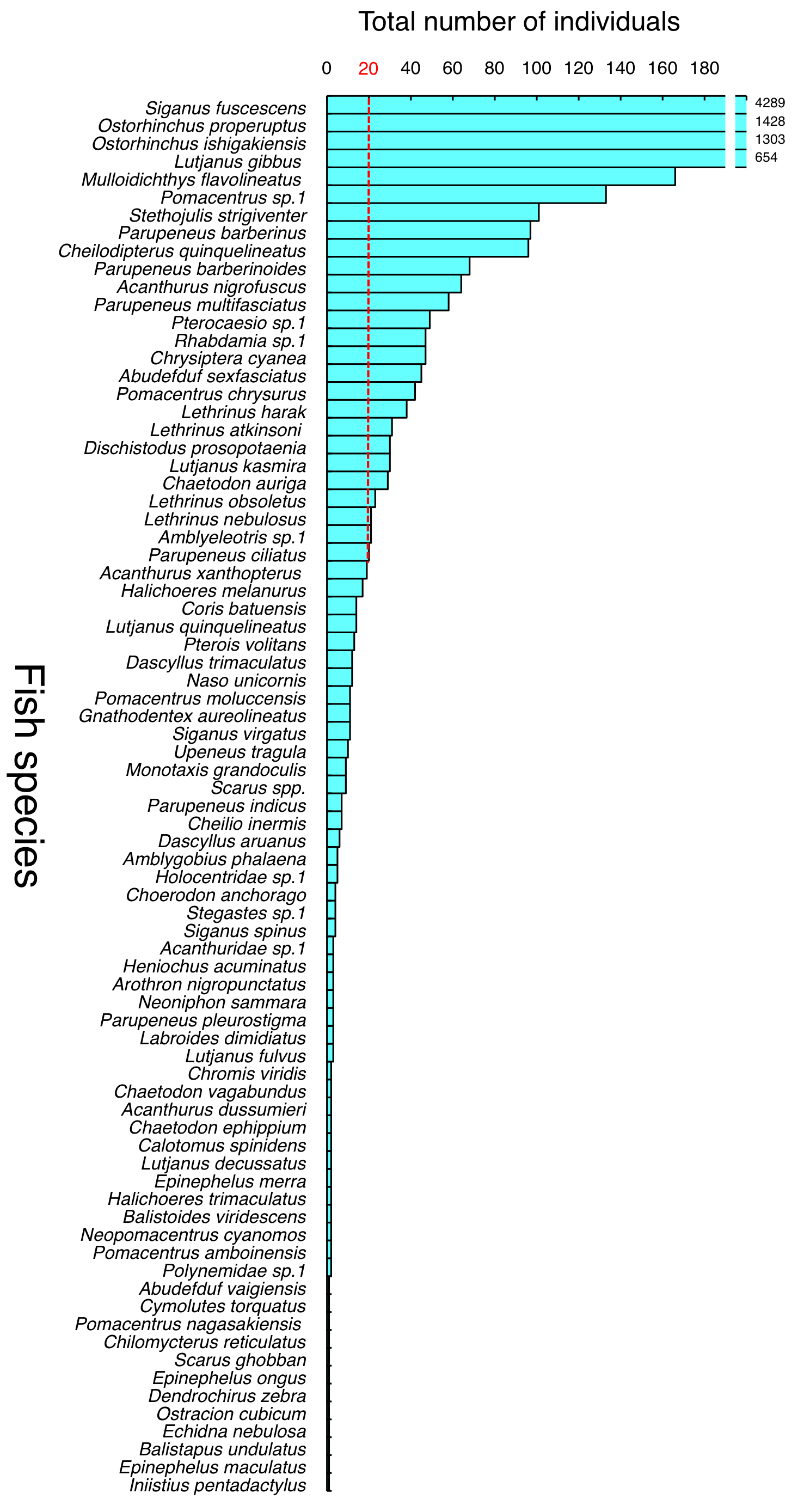

Supplement: Supplemental Information 1 — Red lines represent thresholds between dominant (over 20 individuals) and non-dominant species (less 19 individuals). [file peerj-10-14466-s001.png]
